# The Efficacy and Usability of an Unguided Web-Based Grief Intervention for Adults Who Lost a Loved One During the COVID-19 Pandemic: Randomized Controlled Trial

**DOI:** 10.2196/43839

**Published:** 2023-04-06

**Authors:** Alejandro Dominguez-Rodriguez, Sergio Sanz-Gomez, Leivy Patricia González Ramírez, Paulina Erika Herdoiza-Arroyo, Lorena Edith Trevino Garcia, Anabel de la Rosa-Gómez, Joel Omar González-Cantero, Valeria Macias-Aguinaga, Melina Miaja

**Affiliations:** 1 Department of Psychology, Health and Technology University of Twente Enschede Netherlands; 2 Health Sciences Area Valencian International University Valencia Spain; 3 Department of Psychiatry Universidad de Sevilla Seville Spain; 4 School of Medicine and Health Sciences Tecnologico de Monterrey Guadalajara Mexico; 5 School of Psychology Universidad Internacional del Ecuador Quito Ecuador; 6 Faculty of Higher Studies Iztacala National Autonomous University of Mexico State of Mexico Mexico; 7 Department of Behavioral Sciences Centro Universitario de los Valles Universidad de Guadalajara Guadalajara Mexico

**Keywords:** web-based intervention, usability, complicated grief, hopelessness, suicidal risk, depression, anxiety, posttraumatic stress, mobile phone

## Abstract

**Background:**

The death of a loved one was a challenge many people faced during the COVID-19 pandemic within the context of extraordinary circumstances and great uncertainty. Grief is an unavoidable part of life, and for most people, feelings of grief decrease naturally over time. However, for some people, grieving can become a particularly painful process with clinical symptoms that may require professional help to resolve. To provide psychological support to people who had lost a loved one during the COVID-19 pandemic, an unguided web-based psychological intervention was developed.

**Objective:**

The main objective of this study was to evaluate the efficacy of the web-based treatment, Grief COVID (Duelo COVID in Spanish; ITLAB), in reducing clinical symptoms of complicated grief, depression, posttraumatic stress, hopelessness, anxiety, and suicidal risk in adults. The secondary aim was to validate the usability of the self-applied intervention system.

**Methods:**

We used a randomized controlled trial with an intervention group (IG) and a waitlist control group (CG). The groups were assessed 3 times (before beginning the intervention, upon completing the intervention, and 3 months after the intervention). The intervention was delivered on the web in an asynchronous format through the Duelo COVID web page. Participants created an account that could be used on their computers, smartphones, or tablets. The evaluation process was automated as part of the intervention.

**Results:**

A total of 114 participants were randomly assigned to the IG or CG and met criteria for inclusion in the study (n=45, 39.5% completed the intervention and n=69, 60.5% completed the waitlist period). Most participants (103/114, 90.4%) were women. The results indicated that the treatment significantly reduced baseline clinical symptoms in the IG for all variables (*P*<.001 to *P*=.006), with larger effect sizes for depression, hopelessness, grief, anxiety, and risk of suicide (all effect sizes ≥0.5). The follow-up evaluation showed that symptom reduction was maintained at 3 months after the intervention. The results from the CG showed that participants experienced significantly decreased levels of hopelessness after completing the time on the waitlist (*P*<.001), but their suicidal risk scores increased. Regarding the usability of the self-applied intervention system, the results indicated a high level of satisfaction with the Grief COVID.

**Conclusions:**

The self-applied web-based intervention Grief COVID was effective in reducing symptoms of anxiety, depression, hopelessness, risk of suicide risk, posttraumatic stress disorder, and complicated grief disorder. Grief COVID was evaluated by the participants, who reported that the system was easy to use. These results affirm the importance of developing additional web-based psychological tools to help reduce clinical symptoms in people experiencing grief because of the loss of a loved one during a pandemic.

**Trial Registration:**

ClinicalTrials.gov NCT04638842; https://clinicaltrials.gov/ct2/show/NCT04638842

## Introduction

### Overview

According to the World Health Organization [1], as of July 2022, there had been 6,343,783 deaths worldwide caused by the COVID-19 [1]. In the American continent, 2,754,186 deaths were reported (Pan American Health Organization) [2], including 325,976 deaths reported in Mexico [3]. In addition to the high mortality rates during the pandemic, other factors, such as the risk of contagions, social isolation, and economic insecurity, precipitated mental health problems, including anxiety, stress, depression, posttraumatic stress symptoms [4-7], and an increased probability of developing complicated grief disorder (CGD) [8]. According to Wallace et al [9], the “COVID-19 pandemic has disrupted usual experiences of grief, and modifications of approaches to support grief are needed.” There is scarce evidence at this time about mental health interventions during the COVID-19 pandemic or about interventions specifically related to grief [10]. Web-based interventions can enhance care coverage while maintaining the same quality of care and efficacy. Interventions delivered on the web allow greater accessibility, flexibility, availability, scalability, and reliability, constituting a viable alternative to face-to-face mental health services [11].

### Background

Grief is a natural reaction to loss, often caused by the death of a loved one. Grief helps individuals adapt to the new reality [10]. Grief is an inevitable and painful experience in life with affective, cognitive, behavioral, and spiritual manifestations [4,12]. Some of the most common symptoms include physical distress, sadness, anxiety, confusion, longing, rumination about the past, and fear about the future. People might also experience regret for something lost, remorse for something that occurred in the past, or sorrow for a mishap [13]. Manifestations and duration of grief vary widely, but symptoms are generally expected to improve within the first 2 to 4 months. Culture can affect the grieving period, mediating the relationship between grief and time to heal [4,13]. Other factors that can affect a person’s experience of grief are individual characteristics and the type of relationship they had with the deceased person, making the process more challenging to overcome [8].

There is an essential difference between grief and mourning. Grief refers to the reaction to loss, whereas mourning refers to the adaptation process of loss [14]. Mourning is an important protective factor against pathological grief [12]. CGD, also called persistent grief or persistent complex bereavement disorder, is characterized by persistent (lasting >6 months) and intense grief symptoms. This disorder can impede the return to everyday life and cause significant distress or impairment in functioning [8,10,15,16]. Hopelessness, rumination, alienation, depression, anxiety, yearning, guilt, anger, denial, blame, shame, or posttraumatic stress symptoms may be present when experiencing CGD [10,12,17,18].

CGD is more likely to occur when the bereaved person does not have social support [15], does not go through the normal grieving process, and cannot return to their everyday life and when symptoms of grief persist. Furthermore, the process of mourning may be interrupted for various reasons, for example, if the individual was not able to attend the deceased person’s funeral, if they do not entirely accept or they deny the loss, if the death was unexpected or violent, and if the individual experienced multiple losses at once or over a short period of time. In addition, people with a history of mental health disorders (eg, mood disorders or addiction) are more likely to develop CGD [4,12].

Before the COVID-19 pandemic, Lundorff et al [19] reported a CGD prevalence of 10% in bereaved adults in a systematic review. As losses owing to COVID-19 meet all the risk factors for traumatic grief [20,21], an increase in the prevalence of CGD was expected. Tang and Xiang [17] reported a CGD prevalence of 37.8% in 476 participants mourning losses owing to COVID-19, with data collected from September 1, 2020, to October 3, 2020. In turn, secondary and concurrent factors can make grief more difficult to overcome at this time, such as unexpectedly high mortality rates worldwide, isolation measures to prevent infections, intrahousehold tensions, worries about possible contagion and the availability of health care, the absence of farewell rituals, or the stigma surrounding COVID-19 patients and their families. These circumstances have generated instability in many domains, including social networks and relationships, finances, work, autonomy, safety, social roles, plans, recreation, and personal freedom, among others [4,8-10,13,22-25]. In the face of these deaths and secondary losses, the need for interventions to treat grief is evident [4].

Laranjeira et al [10] reviewed grief support programs and bereavement interventions in response to the COVID-19 pandemic. Most interventions and programs were implemented in a clinical setting by health professionals. The interventions included support via telephone, psychoeducation, counseling, psychological support, and specialized mental health care cognitive behavioral therapy (CBT) interventions. Proposals for grief interventions during the COVID-19 pandemic [14,26-29] are scarce, and in some cases, the results on their effectiveness are yet to be published. A small number of studies have provided results with a limited sample size of 4 participants [29].

There are initial studies that have been applied to people who are experiencing grief. For example, Reitsma et al [16] designed a web-based intervention based on CBT to reduce CGD, posttraumatic stress, and symptoms of depression for adults who had lost a loved one during the COVID-19 pandemic at least 3 months before participating in the study. The treatment consisted of 8 sessions delivered over a maximum of 12 weeks. The intervention included psychoeducation, exposure, and cognitive-restructuring assignments. Subsequently, in the second part of the intervention, trained psychologists guided the participants via email. The results of this study have recently been published [27], involving 65 Dutch adults experiencing bereavement during the pandemic. The study showed the effectiveness of this web-based treatment in reducing disturbed grief, posttraumatic stress, and depression symptoms.

In another study, Tang et al [28] developed a treatment to help Chinese people who were experiencing CGD. The treatment focused on meaning reconstruction and CBT for complicated grief through imaginal exposure, cognitive restructuring, and behavioral activation. This intervention included 8 to 10 sessions and was preceded by training with professional counselors. However, these results have not yet been reported.

Another study by Borghi et al [26] reported their experience with a phone-based early psychological intervention conducted by the clinical psychology unit of an Italian hospital. The psychologists worked with individuals via phone who had lost a loved one to COVID-19. The intervention involved 3 phases: the management of practical procedures related to death, the primary preventive phone-based psychological intervention, and a community-based psychotherapeutic intervention for those who were determined to be at risk for prolonged or excessive grief. The last group was offered individual psychotherapeutic grief sessions via video calls. Menichetti et al [30] published a qualitative study on this intervention. The study included 246 families and examined their experiences and needs regarding the grieving process and intervention and the role that the psychologists played through the phone calls. A thematic analysis was performed using transcripts of the calls, semistructured interviews with the psychologists involved, and observation of psychologists’ peer group discussions. The findings suggest that psychologists can fulfill both social-institutional and psychological-human functions to support individuals in dealing with reactions to the loss of a family member during the COVID-19 pandemic.

Finally, Tur et al [29] used a multiple-baseline single-case experimental design to evaluate a web-based CBT program (GROw) for adults with CGD. The program involved behavioral activation therapy and mindfulness. Only 6 participants were recruited; of these, 2 did not complete the intervention. The results of the study were inconclusive; only half of the participants who finished the treatment demonstrated a clinically significant change in the bereavement measures.

### Theoretical Framework for the Intervention

The intervention Grief COVID (ITLAB) draws on the principles of Worden’s [31] Four Tasks of Grieving. Worden [31] suggests that there are 4 tasks that one must accomplish for equilibrium to be reestablished. The first task is to accept the reality of the loss (intellectual acceptance and emotional acceptance). The second task is to work through the pain of grief, including various emotions such as sadness, fear, loneliness, despair, hopelessness, anger, guilt, blame, shame, relief, and other emotions associated with the loss. The third task is to adjust to living in the environment where the deceased person is no longer there. Finally, the fourth task is to establish an appropriate, ongoing emotional connection with the person who has died, and this also permits one to continue living.

In this regard, Ireland implemented a National Bereavement Support line in response to the COVID-19 pandemic. The service was based on Psychological First Aid principles adapted to integrate bereavement education based on 2 models: Worden’s Task Approach and Stroebe and Schut’s Dual Process Model. The findings from the evaluation of the service suggest that Psychological First Aid is an effective first-line intervention in response to the COVID-19 pandemic. The program provides a supportive, compassionate listening service; educational resources; and referrals to community services [32].

To the authors’ best knowledge, in Latin America, no web-based interventions have been developed before our study to support individuals going through a grieving process. Clinical symptoms and variables of well-being were assessed as proposed in the Grief COVID protocol article [11]. The results have been organized into 2 manuscripts that include different variables. One article has been submitted and is under review for publication, and the second constitutes the present manuscript.

### Aims

This study aimed to present the efficacy of Grief COVID (Duelo COVID in Spanish) in decreasing the risk of developing CGD and in reducing the symptoms of anxiety, depression, hopelessness, and posttraumatic stress disorder (PTSD) and the risk of suicide. The secondary objective was to validate the usability of the self-applied intervention system.

### Hypotheses

#### Primary Hypothesis

Participants in the self-applied multicomponent web-based psychological intervention would report better indicators related to grief and show a reduction in symptoms of anxiety, depression, PTSD, and hopelessness and the risk of suicide compared with the waitlist group. These changes would be maintained for 3 months after treatment completion.

#### Secondary Hypothesis

The self-applied multicomponent web-based psychological intervention for the prevention of CGD would show a good level of acceptance or satisfaction or usability measures.

## Methods

### Study Design

The researchers aimed to conduct a randomized controlled trial (RCT), and this study followed the CONSORT (Consolidated Standards of Reporting Trials) and SPIRIT (Standard Protocol Items: Recommendations for Interventional Trials) guidelines. The CONSORT-EHEALTH (Consolidated Standards of Reporting Trials of Electronic and Mobile Health Applications and Online Telehealth) checklist V1.6.2 is provided (Multimedia Appendix 1). The trial registration number of the study is ClinicalTrials.gov NCT04638842. To access the intervention, participants were required to create an account on the Duelo COVID web page [33], which was accessible on computers, smartphones, and tablets. The evaluation process was automated as part of the intervention. The participants received an email confirming their accounts and granting them access to the web page with the informed consent form and intervention materials. Once the participants had completed the initial assessment, those who met the inclusion criteria (and none of the exclusion criteria) were randomly assigned to either the intervention group (IG) or the control group (CG). The participants assigned to the CG did not receive the intervention immediately; instead, they were placed on a waitlist for 36 days. After completing this period, the participants were granted access to the intervention. A permuted blocks algorithm was performed using the Study Randomizer software (Phase Locked Software) [34]. Participants assigned to the IG received an email notifying them that they were able to access the treatment, whereas participants who were assigned to the CG received an email informing them that they would be placed on a waitlist before being able to access the treatment. After 36 days, the participants in the CG received an email notifying them that they would now have access to the treatment after responding to another assessment. More details can be found in a study by Dominguez-Rodriguez et al [11].

### Participants

Participants were recruited primarily through social media networks and enrolled in the Grief COVID platform between December 22, 2020, and November 17, 2021. Participants were included if they met the following criteria:≥18 years of age; internet access; a valid email address; basic digital skills; fluency in Spanish; experience of the loss of a loved one within the last 6 months; and reported symptoms of depression, anxiety, or stress (ie, total scores of the scales >0). Participants were excluded if, at the time of the study, they met one or more of the following criteria: they had a diagnosis of a psychotic disorder; >6 months had passed since the loss of their loved one; they received psychological and pharmacological treatment at the time of the study; and they had a moderate to a high score on the suicide risk scale, a recent suicide attempt (<3 months before the assessment), or a diagnosis of PTSD. Contact information for external psychological services was provided on the platform, giving preference to the services that were free of charge.

### Intervention

Grief COVID is a free, web-based, self-applied, and multicomponent intervention consisting of 12 modules developed by psychologists with broad experience in clinical and research settings and most of them with a PhD degree. It was created using a user experience design and was incorporated with techniques based on CBT [35], behavioral activation therapy [36], mindfulness [37], and positive psychology [38]. The intervention consists of 12 sessions, delivered over a period of 36 days. Each session was delivered in 2 formats, video or text, and the participants could choose either format. Upon completing each module, the participants were presented with a 5-question quiz. Participants were required to answer at least 60% (3/5) of the questions correctly to progress to the next module. Once a passing score was achieved, a message appeared informing the user that after a 3-day period, the next module would be available. Participants were recommended to progress through 2 modules per week or 1 every 3 days. A detailed description of the intervention is available in the protocol article [11]. The web-based intervention can be accessed on the website [33]. The main web page displays the institutions and universities that made this intervention possible. Sample screenshots of the web-based intervention are presented in Multimedia Appendix 2.

### Measures

The primary outcomes were related to grief, depression, PTSD, hopelessness, anxiety, and suicide risk. The secondary outcome was linked to the participant’s perception of the platform’s usability.

The following instruments were used:

1. *Inventory of Complicated Grief* [39]: This is composed of 19 items, with a 5 Likert-type scale ranging from 0 to 4, where 0=*never*, 1=*rarely*, 2=*sometimes*, 3=*often*, and 4=*always*. The items assess the frequency of each type of symptom (emotional, cognitive, or behavioral). The scores fluctuate between 0 and 76 points. Scores >25 are indicative of complicated grief. The properties of the scale adaptation to Spanish have good internal consistency results (Cronbach α=.88). The version of Limonero García et al [40] was used in this study.
2. *Center for Epidemiologic Studies Depression Scale* [41]: This evaluates depression levels in the past 2 weeks. It contains 20 questions and 4 possible answers: rarely or never (<1 day), sometimes or rarely (1-2 days), occasionally or a good part of the time (3-4 days), and most of the time (5-7 days). This instrument has been validated in the Mexican population (Cronbach α>.90) [42].
3. *Posttraumatic Stress Disorder Symptom Scale* (PSS) [43]: This is a 17-item scale. The interviewer uses a 4-point scale: 0=*not at all*, 1=*a little bit*, 2=*somewhat*, and 3=*very much*. The maximum possible score is 51 (severely affected), and the minimum possible score is 0 (total absence of symptoms). The diagnosis of PTSD is made when 1 symptom of re-experience, 3 symptoms of avoidance, and 2 symptoms of activation are observed [43]. The validated version in Spanish was used in this study [44].
4. *Beck Hopelessness Scale* [45]: This comprises 20 items with a true or false response option. The scores range from 0 to 20, with higher scores indicating a higher level of hopelessness. It is a widely validated and used scale. For this study, the version validated in the Mexican population was applied [46].
5. *Generalized Anxiety Disorder-7* [47]: This is a brief scale consisting of 7 items designed to measure the severity of symptoms of generalized anxiety disorder during the last 2 weeks. A Likert format is used, with scores from 0 to 3, and the maximum total score is 21. A score between 0 and 4 indicates that anxiety is not perceived, and a score between 15 and 21 indicates severe perceived anxiety. The Spanish version by García-Campayo et al [48] was used in this study.
6. *Plutchik Suicide Risk Scale* (PSRS) [49]: This assesses the risk of suicide through questions posed dichotomously (yes or no), and a history of suicide attempts, suicidal ideation, and suicide plans are considered. This scale establishes a cutoff point of >6 that differentiates people at risk of suicide from those who are not. It has good reliability (Cronbach α=.74) and has been used in previous studies of the Mexican population [50].
7. *System Usability Scale* (SUS) [51]: This validates the usability of a system. It is composed of 10 items, which are answered on a 5-point Likert-type scale concerning the degree to which the respondent agrees with each statement (1=*strongly disagree* to 5=*strongly agree*). To obtain the global score of this scale, all the values obtained must be added and multiplied by 2.5, resulting in a number between 0 and 100, which indicates the global value of this scale. Sociodemographic data such as age, gender, country, educational level, and employment status were requested.

### Statistical Analyses

Data analyses were performed using SPSS (version 26; IBM Corp). The normality of the data was examined using the Kolmogorov-Smirnov test. None of the variables assessed in this study followed normal distribution. Therefore, nonparametric tests were used.

Sociodemographic characteristics of the sample between groups (IG vs CG) were explored using the Mann-Whitney *U* test (for participants’ age) and Pearson *χ*^2^ test (for comparisons by gender, country, working status, and educational level). Within-subject comparisons (pretest, posttest, and follow-up) were calculated using the Wilcoxon signed-rank test. For statistically significant changes, Hedges *g* was used to measure the effect sizes. Finally, the participants who completed the intervention filled out a brief questionnaire to share their opinions about the usability of the treatment. For each question, the mean, SD, and range were calculated.

The final sample size obtained for this study was greater than that necessary for the a priori power analysis performed. This was performed using G*Power software (Universität Düsseldorf) with the following parameters: mixed between-within-groups ANOVA tests, Cronbach α=.05, and Cohen *d*=0.8 [11]. However, owing to the high dropout rate, the valid sample for the estimation of treatment effectiveness at follow-up was compromised. Thus, a post hoc sensitivity analysis was performed for the 18 participants at follow-up: Wilcoxon signed-rank test (matched pairs), Cronbach α=.05 (bilateral), and effect size=0.93.

### Ethics Approval

The Research Ethics Committee of the Autonomous University of Ciudad Juárez, Mexico, approved the study (approval ID: CEI-2020-2-226). Participation was voluntary, and all the participants provided written informed consent to participate in this study and for their data to be used for research purposes. The study protocol is available in a study by Dominguez-Rodriguez et al [11].

## Results

### Characteristics of the Participants and Dropout Rates

**Figure 1:**
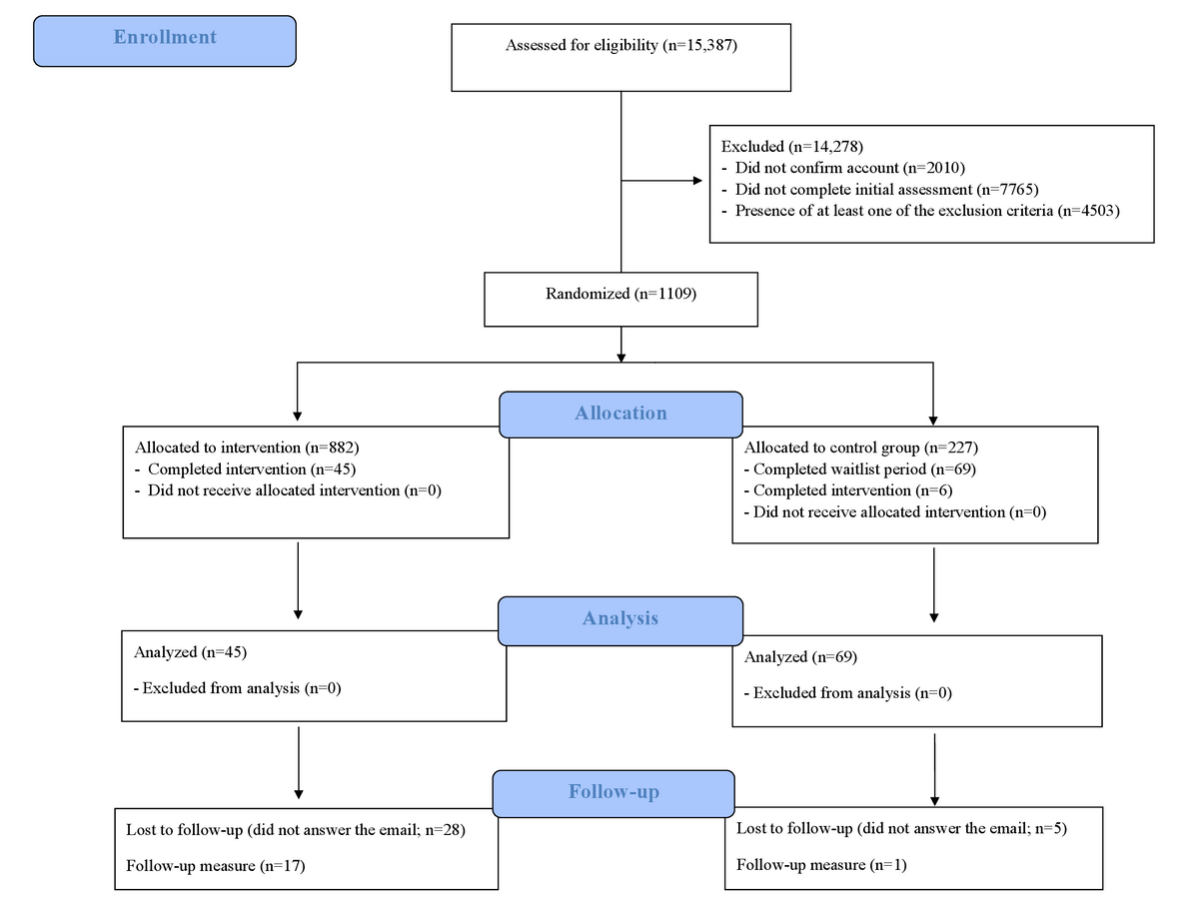
CONSORT (Consolidated Standards of Reporting Trials) flow diagram of the study design.

A total of 15,387 participants were assessed for eligibility. Of the 15,387 participants, 14,278 (92.79%) were excluded from the study. The remaining 7.21% (1109/15,387) of participants were randomly assigned to the IG (n=882, 79.53%) and CG (n=227, 20.47%). Ultimately, 10.28% (114/1109) of participants completed the conditions (n=45, 39.5% completed the intervention, and n=69, 60.5% completed the waitlist period; Figure 1). The trial ended when the proposed sample size was exceeded, and the follow-up period was also completed.

Most participants were from Mexico (110/114, 96.5%). In all, 90.4% (103/114) were women, 8.8% (10/114) were men, and 0.9% (1/114) was a nonbinary person. Two-thirds of the participants (77/114, 67.5%) reported being actively working. Educational attainment was high, with 61.4% (70/114) of participants reporting university degrees, 21.1% (24/114) reporting a master’s degree, 14% (16/114) reporting a high school diploma, 1.8% (2/114) reporting a PhD, and the remaining 2 (1.8%) reporting other educational levels.

A total of 39.5% (45/114) of participants received treatment, and 60.5% (69/114) of participants were assigned to the waitlist CG. The sociodemographic characteristics of both groups were similar, with the exception of age (Multimedia Appendix 3).

A large number of participants (treatment condition: 576/882, 65.3%; CG condition: 49/69, 71%) dropped out of the intervention before beginning, without completing any of the modules. A considerable dropout rate was observed among the participants in module 1 (112/882, 12.7% of participants from the IG). More details are provided in Table 1.

**Table 1:** Detailed dropout rates by participants and conditions.

Last module accessed	Treatment participants (n=882), n (%)	Control participants (n=69), n (%)
Assigned to the intervention but did not access any module	576 (65.3)	49 (71)
1	112 (12.7)	3 (4.3)
2	57 (6.5)	3 (4.3)
3	18 (2)	4 (5.8)
4	21 (2.4)	1 (1.4)
5	11 (1.2)	0 (0)
6	9 (1)	2 (2.9)
7	10 (1.1)	0 (0)
8	4 (0.5)	0 (0)
9	6 (0.7)	0 (0)
10	9 (1)	0 (0)
11	4 (0.5)	1 (1.4)
12	45 (5.1)	6 (8.7)

The mean age of the treatment group was 45.7 (SD 10.5) years, with participants’ ages ranging from 22 to 62 years. In contrast, the mean age for the CG was 34.4 (SD 10.4) years, ranging from 21 to 58 years.

### Comparison of Clinical Symptoms Between Groups at the Pretest

Table 2 depicts the sample clinical characteristics at the pretest assessment. No statistically significant differences were found between the IG and CG, with the exception of a higher score for PTSD symptoms in the CG.

**Table 2:** Dependent variables at the pretest measured by study condition.

Instrument	Participants, n (%)		Median (IQR)		*P* value^a^
	Control (n=69)	Treatment (n=45)	Control	Treatment	
ICG^b^	69 (100)	45 (100)	22 (18-32)	12 (11.5-17)	.15
CESD-R^c^	63 (91)	41 (91)	15 (11-22)	17 (10-24)	.48
PSS^d^	69 (100)	45 (100)	13 (9-15)	9 (6-13)	.*003*^e^
BHS^f^	69 (100)	45 (100)	3 (2-6)	3 (1-5)	.33
GAD-7^g^	69 (100)	45 (100)	6 (4-10)	7 (4-8)	.99
PSRS^h^	69 (100)	45 (100)	3 (2-5)	3 (1-6)	.85

^a^*P* value for the Mann-Whitney *U* test.

^b^ICG: Inventory of Complicated Grief.

^c^CESD-R: Center for Epidemiologic Studies Depression Scale.

^d^PSS: Posttraumatic Stress Disorder Symptom Scale.

^e^Significant *P* value.

^f^BHS: Beck Hopelessness Scale.

^g^GAD-7: Generalized Anxiety Disorder-7.

^h^PSRS: Plutchik Suicide Risk Scale.

### The Effects of the Intervention Versus the Waitlist on Clinical Symptoms: Comparisons Between Groups

We found pronounced differences between the IG and CG on all the measures examined (Table 3) after the participants had completed the intervention or the waitlist period. Moreover, the gap between the PSS scores between the groups further widened.

**Table 3:** Differences between intervention group and control group comparing treatment versus waiting list.

Instrument	Participants, n (%)			Median (IQR)			*P* value^a^
	Control (n=69)	Treatment (n=45)	Control		Treatment		
ICG^b^	51 (74)	43 (96)	20 (13-31)		10 (5-14)	*<.001* ^c^	
CESD-R^d^	65 (94)	45 (100)	14 (11-17)		6 (4-10)	*<.001* ^c^	
PSS^e^	51 (74)	43 (96)	11 (7-17)		5 (3-9)	*<.001* ^c^	
BHS^f^	51 (74)	43 (96)	1 (0-3)		0 (0-1)	*.005* ^c^	
GAD-7^g^	51 (74)	43 (96)	7 (4-11)		2 (1-5)	*<.001* ^c^	
PSRS^h^	54 (78)	44 (98)	4 (2-5)		2 (1-3)	*<.001* ^c^	

^a^*P* value for the Mann-Whitney *U* test.

^b^ICG: Inventory of Complicated Grief.

^c^Significant *P* values.

^d^CESD-R: Center for Epidemiologic Studies Depression Scale.

^e^PSS: Posttraumatic Stress Disorder Symptom Scale.

^f^BHS: Beck Hopelessness Scale.

^g^GAD-7: Generalized Anxiety Disorder-7.

^h^PSRS: Plutchik Suicide Risk Scale.

### The Efficacy of the Intervention: Changes Within Subjects

The IG showed clinical improvement in all the measures assessed (Table 4). Psychological treatment had larger effect sizes on depression scores (1.3), hopelessness (0.9), grief (0.7), anxiety (0.7), and suicide risk (0.5). To a lesser extent, it also affected posttraumatic stress symptoms (0.3). It is worth highlighting that whereas the IG showed a medium-sized decrease in the PSRS score (ie, a reduction of symptoms), the CG exhibited a statistically significant increase in the suicide scale score after the waitlist period (*P*=.004), albeit small in size. The CG also reported a decrease in hopelessness scores, with a medium-sized effect (0.6).

**Table 4:** Changes in within-subjects measures by study condition.

	Pretest						Posttest measure (intervention vs waiting list)							Hedges *g* (95% CI)					*P* value^a^		
	Participants, n (%)		Median (IQR)			Participants, n (%)				Median (IQR)			Control (n=69)			Treatment (n=45)		Control (n=69)			Treatment (n=45)
	Control (n=69)	Treatment (n=45)	Control (n=69)	Treatment (n=45)	Control (n=69)			Treatment (n=45)	Control (n=69)		Treatment (n=45)										
ICG^b^	69 (100)	45 (100)	22 (18-32)	12 (11.5-17)	51 (74)			43 (96)	20 (13-31)		10 (5-17)	N/A^c^			0.7 (0.4 to 1.1)		.39			*<.001* ^d^	
CESD-R^e^	63 (91)	41 (91)	15 (11-22)	17 (10-24)	65 (94)			45 (100)	14 (11-17)		6 (4-10)	N/A			1.3 (0.9 to 1.8)		.10			<*.001*^d^	
PSS^f^	69 (100)	45 (100)	13 (9-15)	9 (6-13)	51 (74)			43 (96)	11 (7-17)		5 (3-9)	N/A			0.4 (0.1 to 0.8)		.55			*.006* ^d^	
BHS^g^	69 (100)	45 (100)	3 (2-6)	3 (1-5)	51 (74)			43 (96)	1 (0-3)		0 (0-1)	0.6 (0.3 to 0.9)			0.9 (0.5 to 1.4)		*<.001* ^d^			*<.001* ^d^	
GAD-7^h^	69 (100)	45 (100)	6 (4-10)	7 (4-8)	51 (74)			43 (96)	7 (4-11)		2 (1-5)	N/A			0.7 (0.4 to 1.2)		.62			*<.001* ^d^	
PSRS^i^	69 (100)	45 (100)	3 (2-5)	3 (1-6)	54 (78)			44 (98)	4 (2-5)		2 (1-3)	−0.3 (−0.6 to −0.1)			0.5 (0.2 to 0.8)		*.004* ^d^			*.004* ^d^	

^a^*P* value for Wilcoxon signed-rank test.

^b^ICG: Inventory of Complicated Grief.

^c^N/A: not applicable; Hedges *g* values are reported for comparisons with significant *P* values.

^d^Significant *P* values.

^e^CESD-R: Center for Epidemiologic Studies Depression Scale.

^f^PSS: Posttraumatic Stress Disorder Symptom Scale.

^g^BHS: Beck Hopelessness Scale.

^h^GAD-7: Generalized Anxiety Disorder-7.

^i^PSRS: Plutchik Suicide Risk Scale.

### Changes Maintained at Follow-up: The Efficacy After 3 Months

Regarding the 3-month follow-up, all achievements obtained at the posttreatment measure by the IG were sustained (Table 5). Beck Hopelessness Scale, PSS, Generalized Anxiety Disorder-7, and PSRS scores maintained the overall values that reached posttest measures. Center for Epidemiologic Studies Depression Scale and Inventory of Complicated Grief scores decreased further after 3 months. Although some participants were randomly assigned to the waitlist CG and were able to access the intervention after completing the waiting period, a reduced number (6/69, 9%) of the CG participants completed the entire intervention.

**Table 5:** Changes after treatment until follow-up period for the intervention group.

Instrument	Participants (n=18), n (%)	Before treatment, median (IQR)	After treatment, median (IQR)	*P* value^a^	3-month follow-up, median (IQR)	*P* value^b^
ICG^c^	18 (100)	22.5 (15-29)	12 (7-18)	*.001* ^d^	8 (5-10)	*<.001* ^d^
CESD-R^e^	18 (100)	16 (12-20)	6.5 (5-10)	*.001* ^d^	2 (5-8.5)	*.001* ^d^
BHS^f^	18 (100)	9 (7-11)	5 (3-8)	*.008* ^d^	4.5 (2-9)	*.003* ^d^
PSS^g^	18 (100)	4.5 (3-7)	0 (0-1)	*<.001* ^d^	0.5 (0-1)	*<.001* ^d^
GAD-7^h^	18 (100)	5 (4-8)	2.5 (2-4)	*.001* ^d^	4 (1-6)	*.002* ^d^
PSRS^i^	18 (100)	4 (3-6)	1.5 (1-2)	*.001* ^d^	1 (1-3)	*.001* ^d^

^a^*P* value for Wilcoxon signed-rank test between pretreatment and posttreatment measures.

^b^*P* value for Wilcoxon signed-rank tests between pretreatment and 3-month follow-up measures.

^c^ICG: Inventory of Complicated Grief.

^d^Significant *P* values.

^e^CESD-R: Center for Epidemiologic Studies Depression Scale.

^f^BHS: Beck Hopelessness Scale.

^g^PSS: Posttraumatic Stress Disorder Symptom Scale.

^h^GAD-7: Generalized Anxiety Disorder-7.

^i^PSRS: Plutchik Suicide Risk Scale.

### The Usability of Grief COVID Intervention

All 100% (45/45) of participants who completed the treatment filled out the SUS. Overall, reviews were quite positive, with a quarter of participants (11/45, 24%) selecting the highest score (ie, reporting good usability) in all items and two-thirds of participants (30/45, 67%) giving the highest rating to 8 of 10 items (Table 6).

**Table 6:** Results of System Usability Scale (n=45).

	Ranks, median (IQR; range)
1. “I think that I would like to use this system frequently”	5.0 (4.0-5.0; 1.0-5.0)
2. “I found the system unnecessarily complex”^a^	1.0 (1.0-1.0; 1.0-5.0)
3. “I thought the system was easy to use”	5.0 (5.0-5.0; 1.0-5.0)
4. “I think that I would need the support of a technical person to be able to use this system”^a^	5.0 (5.0-5.0; 1.0-5.0)
5. “I found that various functions in this system were well integrated”	5.0 (4.0-5.0; 1.0-5.0)
6. “I thought there was too much inconsistency in this system”^a^	1.0 (1.0-1.0; 1.0-5.0)
7. “I imagine that most people would learn to use this system very quickly”	5.0 (5.0-5.0; 1.0-5.0)
8. “I found the system very cumbersome to use”^a^	1.0 (1.0-2.0; 1.0-5.0)
9. “I felt very confident in using the system”	5.0 (5.0-5.0; 3.0-5.0)
10. “I needed to learn a lot of things before I could get going with this system”^a^	1.0 (1.0-1.0; 1.0-5.0)

^a^Items are negatively worded (ie, lower scores show greater usability).

## Discussion

### Principal Findings

The objective of this study was to present the efficacy of Grief COVID in decreasing the risk of developing CGD and in reducing symptoms of anxiety, depression, hopelessness, PTSD, and risk of suicide.

The findings of this study showed a reduction in depression, hopelessness, grief, anxiety, and suicide risk and a lesser effect on posttraumatic stress scores in the IG. The results were maintained at the 3-month postintervention follow-up. These results reflect a statistically significant difference compared with the CG scores, which showed an increase in suicide risk after the waitlist period.

Proposals for grief intervention during the COVID-19 pandemic are scarce. Tur et al [29] performed an intervention called “The GROw program,” which evaluated depression, symptoms of grief, and typical grief beliefs, along with daily measures of symptom frequency and intensity using the Emotional Monitor App. Of 6 participants, 4 completed the intervention; of them, only 2 obtained a clinically significant change in the bereavement measures. Second, Reitsma et al [27] performed an RCT to examine the short-term effects of an unguided web-based grief-specific CBT in treating persistent complex bereavement disorder, PTSD, and depression symptoms. A total of 65 Dutch adults participated, all of whom had experienced the death of a loved one at least 3 months before the start of the study during the COVID-19 pandemic. Similar to our results, their findings revealed significant effects for reductions in disturbed grief symptoms and moderate between-group effects for PTSD and depression symptoms.

One of the few RCTs that sought to alleviate the psychological distress caused by COVID-19 is a study by Brog et al [52] in Germany. They developed a web-based self-help intervention based on CBT and psychoeducation. Although their study did not show positive effects on the reduction of symptoms of depression, anxiety, and stress, the intervention was found to be suitable for preventive purposes to help individuals cope with psychological distress. The results showed increased emotion regulation and resilience in participants [52]. In most cases, publications on the effectiveness of grief intervention protocols in the context of the COVID-19 pandemic are yet to be published [26,28].

The Grief COVID intervention was designed using techniques that draw on CBT, behavioral activation therapy, mindfulness, and positive psychology. Most studies to date on grief intervention during the COVID-19 pandemic used CBT based on its known effectiveness in treating grief [16,28,29]. These studies also incorporated behavioral activation, and a study conducted by Tur et al [29] included mindfulness activities as well. No studies were found that treated grief using the principles of positive psychology during the COVID-19 pandemic.

Important issues to consider stem from the unique circumstances that people have experienced during the COVID-19 pandemic. For example, face-to-face contact was restricted, and a large part of society was affected by the economic impact of the pandemic. In this context, web-based interventions have a promising potential for several reasons. First, treating people in this way can reduce the time required to access support services. Second, the cost of web-based treatments may be more affordable for much of the population [10]. A review by Harrop et al [53] describes the recommendations for grief intervention during a disaster. The literature highlights the need for increased psychoeducation regarding grief and available support services for bereaved individuals, as well as screening for the risk of CGD, and the provision of professional mental health support for those with a high risk of CGD. These tenets were integrated into our intervention; all participants completed an initial screening on the platform (results of the pre-evaluation questionnaires can be consulted in the study by Dominguez-Rodriguez et al [5]), which then generated a free psychoeducational resource for bereavement during the pandemic and provided additional contact information for relevant mental health services and professionals. The secondary objective of this study was to validate the usability of the self-applied intervention system. Most participants who finished the intervention (41/45, 91%) reported a high level of satisfaction with the platform. The positive ratings of our intervention system may be attributed, in part, to the user-centered design that accounted for the specific needs and characteristics of the intended users [54]. Laranjeira et al [10] suggested an urgent need for evidence to evaluate the feasibility, effectiveness, and acceptability of web-based interventions in conventional bereavement situations, as well as disaster contexts. Our study contributes to the evidence that begins to lessen this knowledge gap.

The results of this study have several implications for health care providers and local governments. First, web-based interventions have been shown to be effective in reducing psychological distress, including the symptoms of grief, depression, anxiety, and posttraumatic stress [27,29,52]. Second, this treatment modality represents a viable option for people who may have difficulty accessing specialized face-to-face care because of a lack of proximity to services where they live or other reasons. In addition, the costs associated with web-based interventions are lower for public institutions when compared with individual face-to-face interventions, as web-based treatments require only the initial economic investment in and maintenance of the platform [10].

This study does have some limitations, the first of which is related to the dropout rate. Only 5.1% (45/882) of the IG participants completed the intervention. It is not uncommon for internet-based and self-administered interventions to have high dropout rates, particularly among participants assigned to the waitlist group. This may be due to reasons such as a preference for face-to-face treatment or lack of internet access or a digital device on which to access the platform. Another consideration is that 12 modules may be too long for a preventive intervention of this type. However, similar intervention proposals have ranged from 8 to 12 sessions [16,28]. To reduce the incidence of dropouts, further studies should send weekly notifications to users to encourage them to continue with the intervention. In addition, a survey might be sent to participants who decide not to complete the entire program to better understand their reasons and improve upon these points in future studies. Specifically, all the participants on the CG condition completed the pre-evaluation questionnaire and then proceeded to complete a waiting period of 36 days. Once they finished the waiting period, they were requested to repeat the evaluation to assess their symptomatology and confirm that they still did not fulfill any exclusion criteria. Once this was achieved, they were granted access to the intervention. However, most participants (63/69, 91%) assigned to this group did not finish the intervention, as only just 6 participants completed the intervention and one of them answered the follow-up measure. Therefore, we did not report postevaluation data from the CG. Future studies should consider ways to encourage adherence to treatment for those assigned to the waitlist, and this could help reduce the dropout rate. Another limitation of this study is that there were substantially more women than men in both the CG and the IG. This phenomenon has been observed in other similar studies [27,29]. An additional limitation of this study is that to date, few studies regarding web-based interventions to prevent CGD exist, and there is little evidence to suggest that an unguided intervention can be as effective as a guided intervention in treating grief. However, there is existing evidence that suggests that for the treatment of mild symptoms of depression [55] or anxiety [56], unguided web-based interventions have similar outcomes to guided interventions, particularly in the medium term (3- and 6-month follow-ups). Nevertheless, further research is needed to understand the mechanisms of change and variables associated with web-based treatment outcomes.

Another limitation of the study is that because the intervention was web-based, unguided, and preventive in nature, we did not consider it ethical to include individuals who reported substantial suicidality (ie, suicidal intent or plans). This limited external validity, particularly by decreasing the proportion of severely depressed participants in the sample. A further limitation is that to evaluate the results of the intervention, only the participants who completed the intervention could access the postmeasurement assessment. Evaluating participants only at the posttreatment point, and not evaluating them during the intervention, limits our knowledge regarding the treatment’s efficacy at different stages of the intervention. Future studies should include assessment measures at several points throughout the intervention to provide a more complete picture of the treatment’s efficacy at different stages. In this regard, we have conducted 2 studies (Dominguez-Rodriguez, A, unpublished data, March 2023) that help us to better understand the reasons some participants continued with the intervention, whereas some others dropped out. In the first study, the participants who dropped out of the intervention were invited to respond to the SUS, along with ad hoc instruments designed to allow us to analyze the participants’ reasons for abandoning the intervention. The study was conducted using 3 psychological intervention platforms that were available during the COVID-19 pandemic: Salud Mental COVID-19 [57], Duelo COVID-19 [33], and Personal COVID-19 [58]. In addition, we conducted a qualitative study with the Grief COVID participants to gather information about their perceptions of the intervention. The study included participants who completed the intervention as well as those who dropped out. In addition, in this qualitative study, we further explored the important harms or unintended effects in each group while observing none.

The results of these studies will be published in the later articles and will help us understand the underlying causes of the dropout rates (Table 1).

Another limitation is that one of the exclusion criteria was that the participants have a diagnosis of a psychotic disorder. However, no specific instrument was included to assess this criterion, apart from asking the participants if they were taking any form of medication, which could indicate if they were consuming an antipsychotic drug. Future studies similar to ours might consider including assessment measures for psychosis as a part of the intervention. For example, Rüegg et al [59] implemented a self-applied intervention similar to Grief COVID and did include these types of assessments.

Regarding the strengths of this study, to the best of our knowledge, this is the first study to publish the results of an intervention aimed at preventing CGD in Latin America. Furthermore, this study is one of a small number of studies that examined grief in the context of the COVID-19 pandemic. In addition, the sample size of our study is sufficiently large to show statistically significant results, and the study included data from 3 time points. The platform was created using principles of responsive design, which permitted the intervention to be accessed on devices of different screen sizes, including cell phones, tablets, and computers [11]. This is one of the few RCTs of web-based interventions developed and delivered in Latin America, where such interventions, although greatly needed, are still scarce [60]. Finally, Grief COVID is the only web-based intervention implemented for bereaved people in the Latin American region during the COVID-19 pandemic.

### Conclusions

The self-applied web-based intervention Grief COVID was efficient in reducing the symptomatology of anxiety, depression, hopelessness, suicide risk, PTSD, and CGD. Grief COVID was evaluated by the participants who answered the system usability questionnaire as easy to use. The results of this study are highly relevant to professionals in other countries who wish to design, develop, and deliver interventions similar to Grief COVID to provide grief-related support to the general population and decrease the risk of developing CGD and associated clinical correlates.

## Appendix

Multimedia Appendix 1 CONSORT-eHEALTH checklist (V 1.6.1).

Multimedia Appendix 2 Sample screenshots of the web-based intervention.

Multimedia Appendix 3 Sociodemographic characteristics of participants.

## Abbreviations

CBT: cognitive behavioral therapy
CG: control group
CGD: complicated grief disorder
CONSORT: Consolidated Standards of Reporting Trials
CONSORT-EHEALTH: Consolidated Standards of Reporting Trials of Electronic and Mobile Health Applications and Online Telehealth
IG: intervention group
PSRS: Plutchik Suicide Risk Scale
PSS: Posttraumatic Stress Disorder Symptom Scale
PTSD: posttraumatic stress disorder
RCT: randomized controlled trial
SPIRIT: Standard Protocol Items: Recommendations for Interventional Trials
SUS: System Usability Scale

## Data Availability

The data sets generated and analyzed during this study are available from the corresponding author on reasonable request.
