# Supplementary material for: The Efficacy and Usability of an Unguided Web-Based Grief Intervention for Adults Who Lost a Loved One During the COVID-19 Pandemic: Randomized Controlled Trial
Source: J Med Internet Res. 2023 Apr 6;25:e43839. doi: 10.2196/43839 (PMC10131766; doi:10.2196/43839)
Supplement: Multimedia Appendix 2 [file jmir_v25i1e43839_app2.pdf]

This is a Multimedia Appendix to a full manuscript published in the J Med Internet Res. For full copyright and citation information see http://dx.doi.org/10.2196/jmir.43839

**Multimedia Appendix 2.** Sample screenshots of the web-based intervention

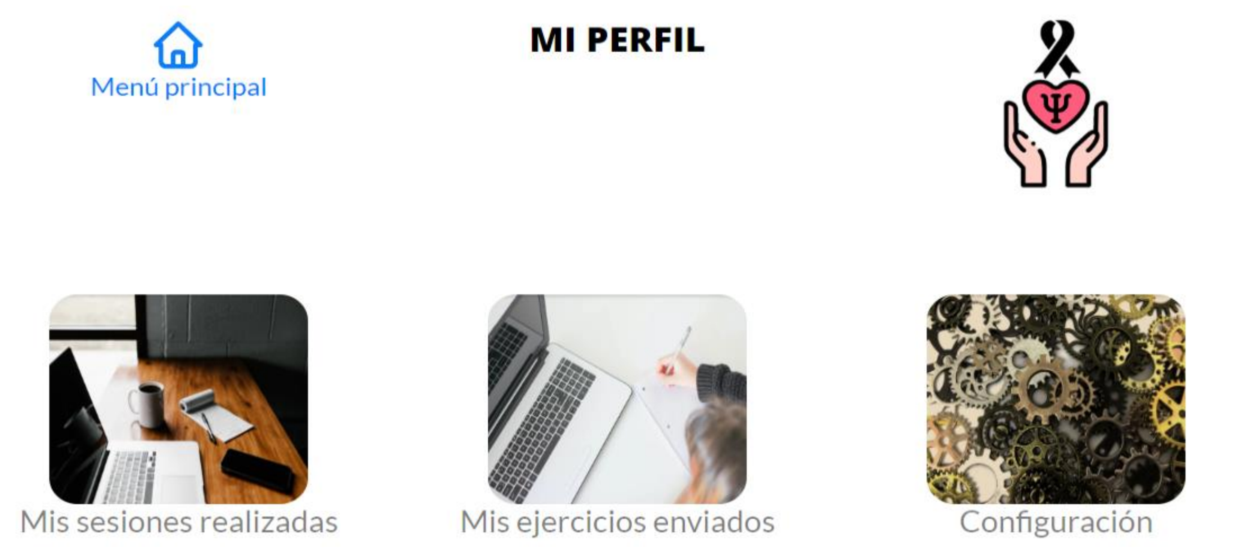


Appendix Figure 1. My profile section. After the user completes each module, there is a three-day interval before the next module is unlocked. Participants receive a reminder via email when they can access the next module. Completed modules are permanently unlocked and can be accessed in the section “My completed sessions” [Mis sesiones realizadas].

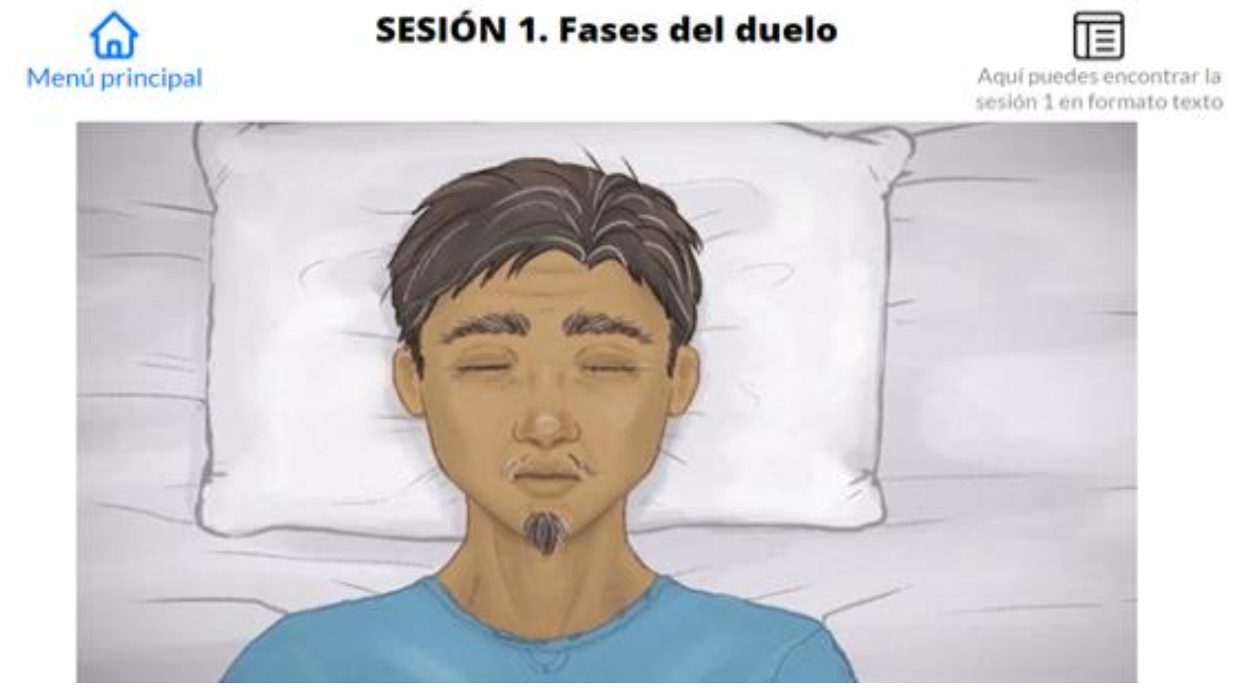


Appendix Figure 2. Screenshot of the first video from Session 1 (Grief phases). The intervention is offered to participants in two formats: 1) animated videos and 2) text. Each session includes examples and activities for participants to put what they learn into practice.

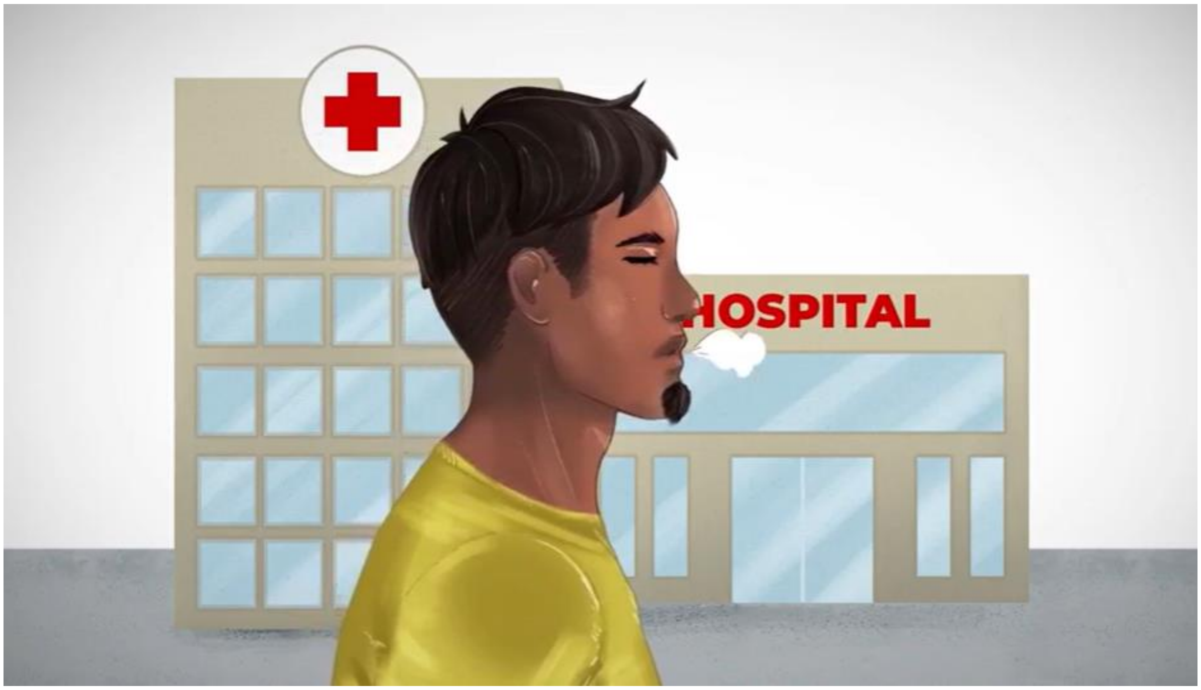


Appendix Figure 3. Screenshot of one of the animated characters from Session 1 (Phases of Grief). The graphic designers created the animations taking into account the diversity of the Mexican population in terms of age, gender, and race/ ethnicity.

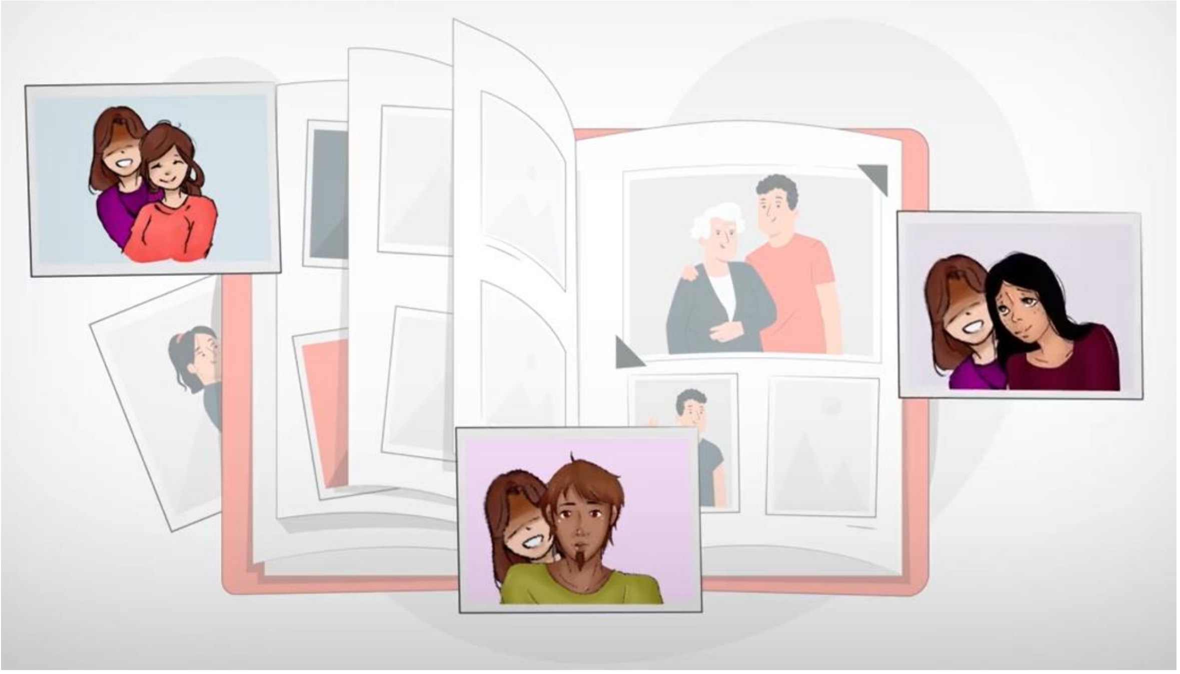

Appendix Figure 4. Screenshot of an activity from Session 2 (Identifying emotions and attending to needs).

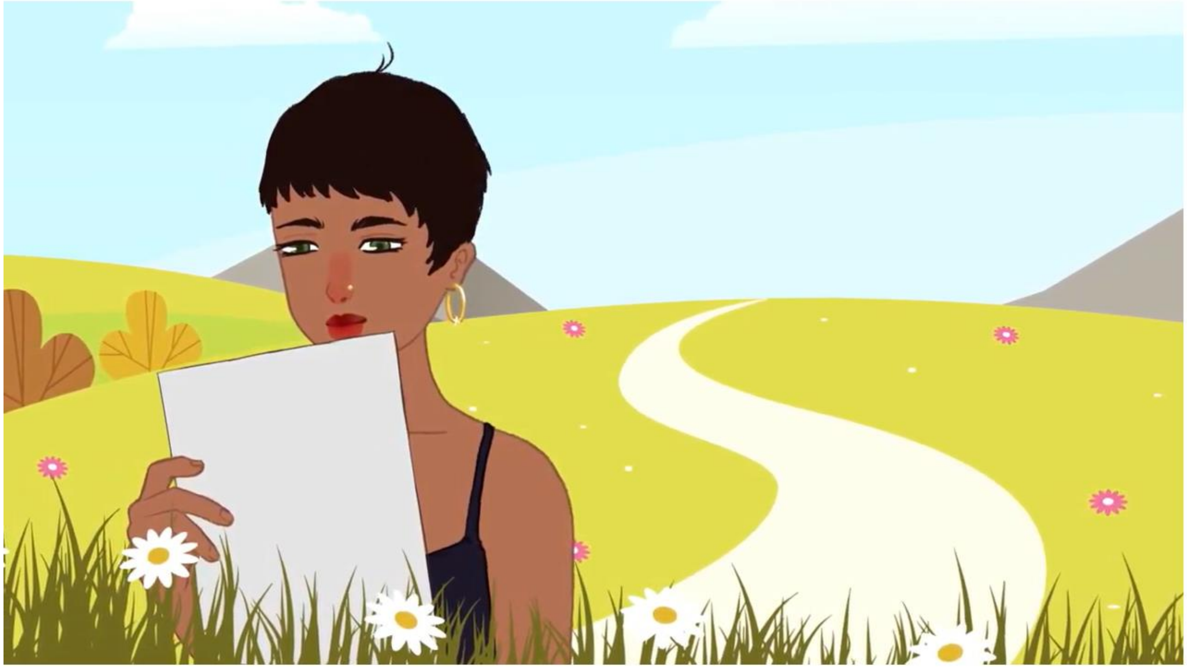

Appendix Figure 5. Screenshot of an animated character from Session 6 (Parting Strategies)

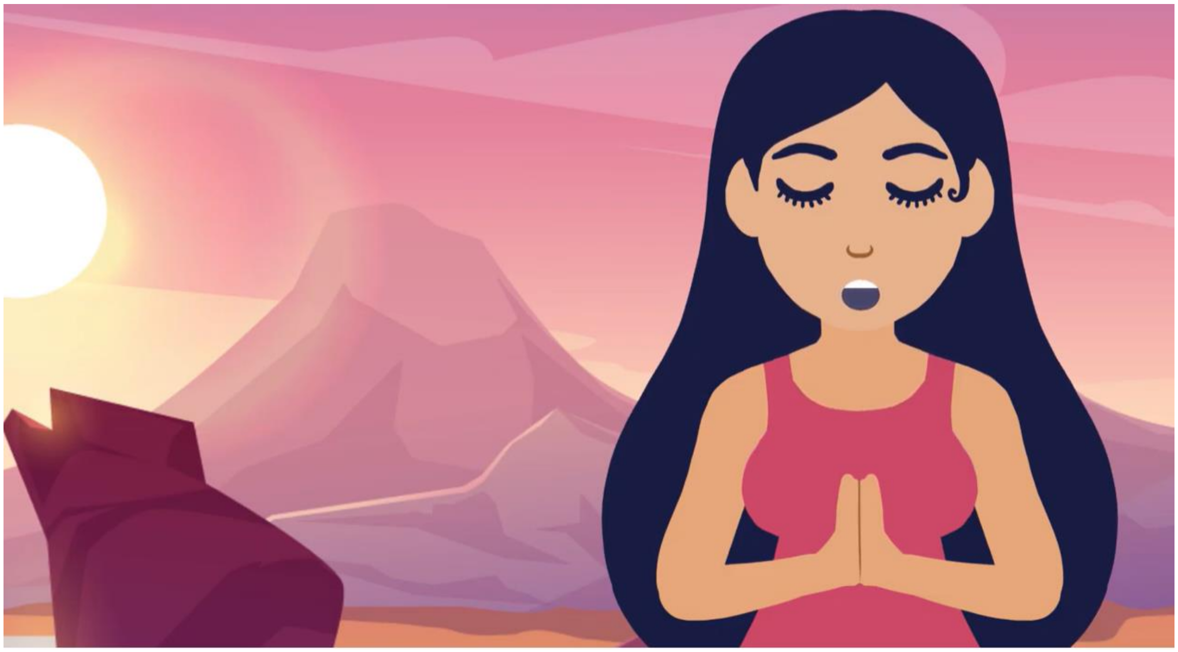

Appendix Figure 6. Screenshot of an animated character from Session 7 (Self-care).

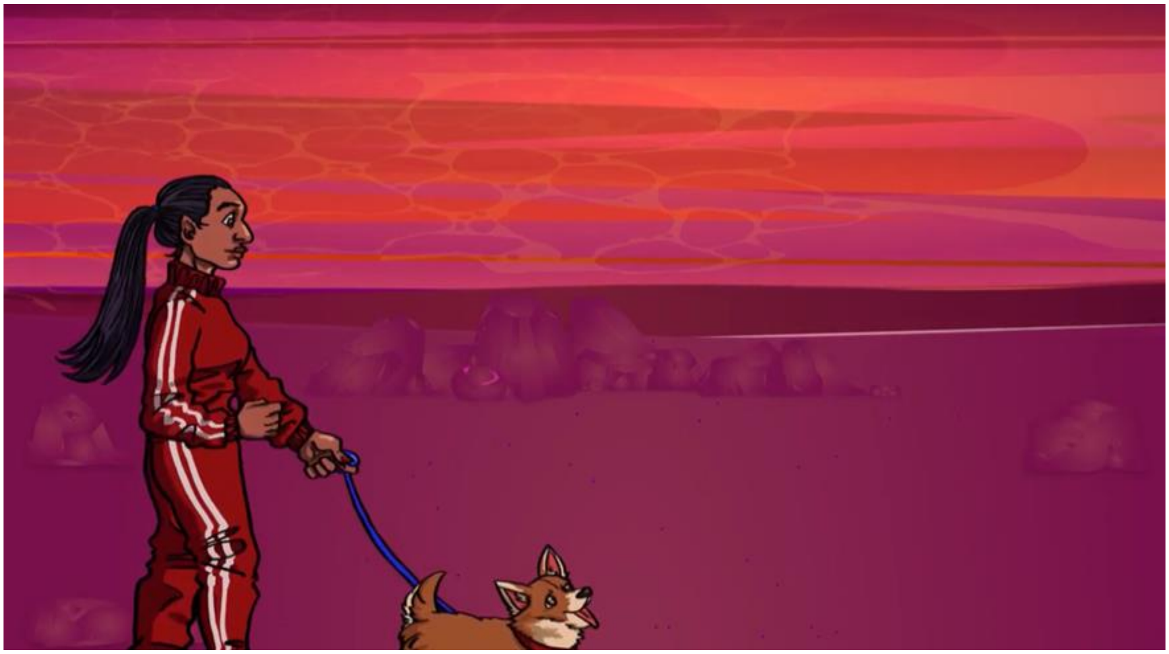

Appendix Figure 7. Screenshot of an animated character from Session 8 (Returning to daily activities).

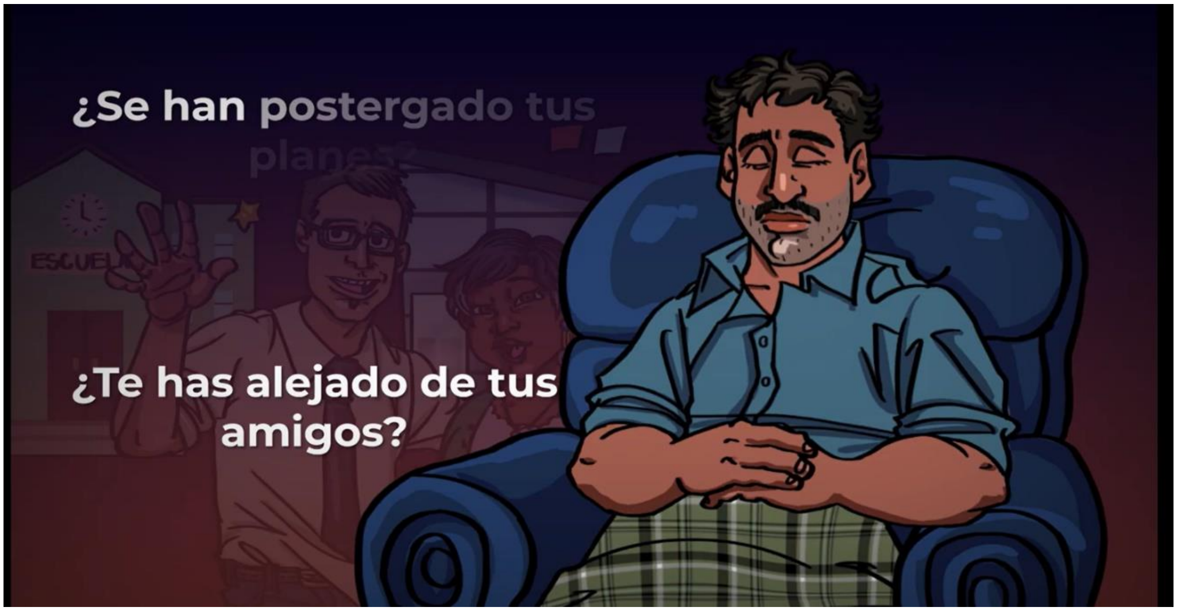


Appendix Figure 8. Screenshot of one of the animated characters from Session 11 (Establishing goals). The text states: Have your plans been postponed? [¿Se han postergado tus planes?], Have you distanced yourself from your friends? [¿Te has distanciado de tus amigos?]

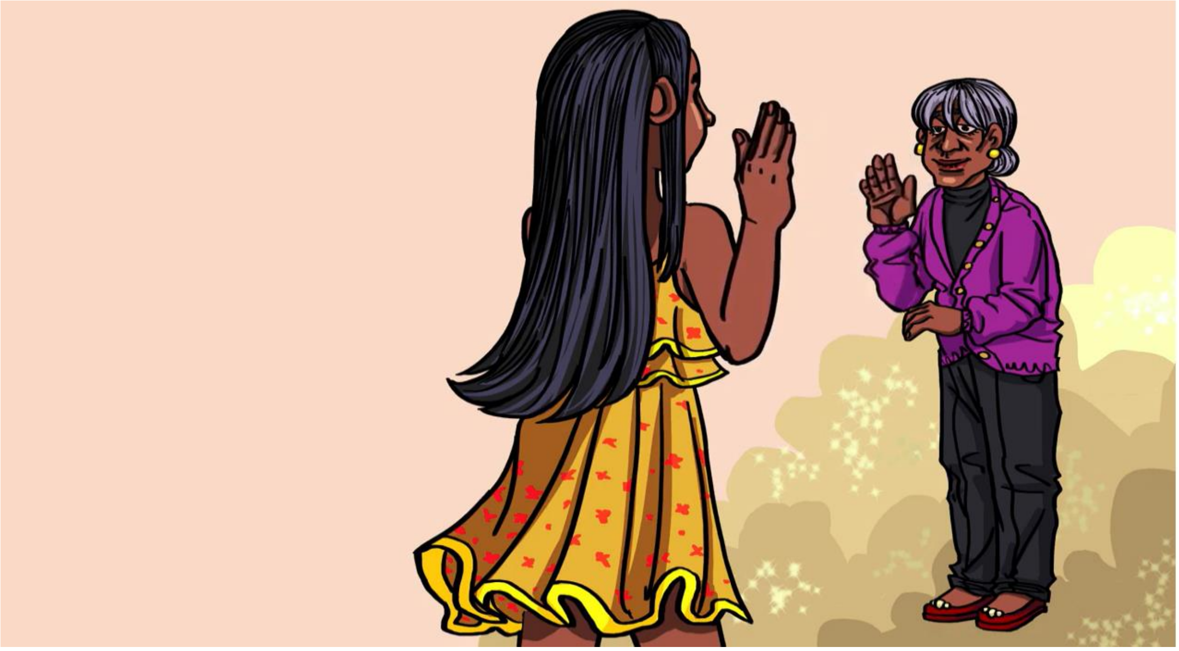

Appendix Figure 9. Screenshot of animated characters from Session 12 (Relapse prevention plan).

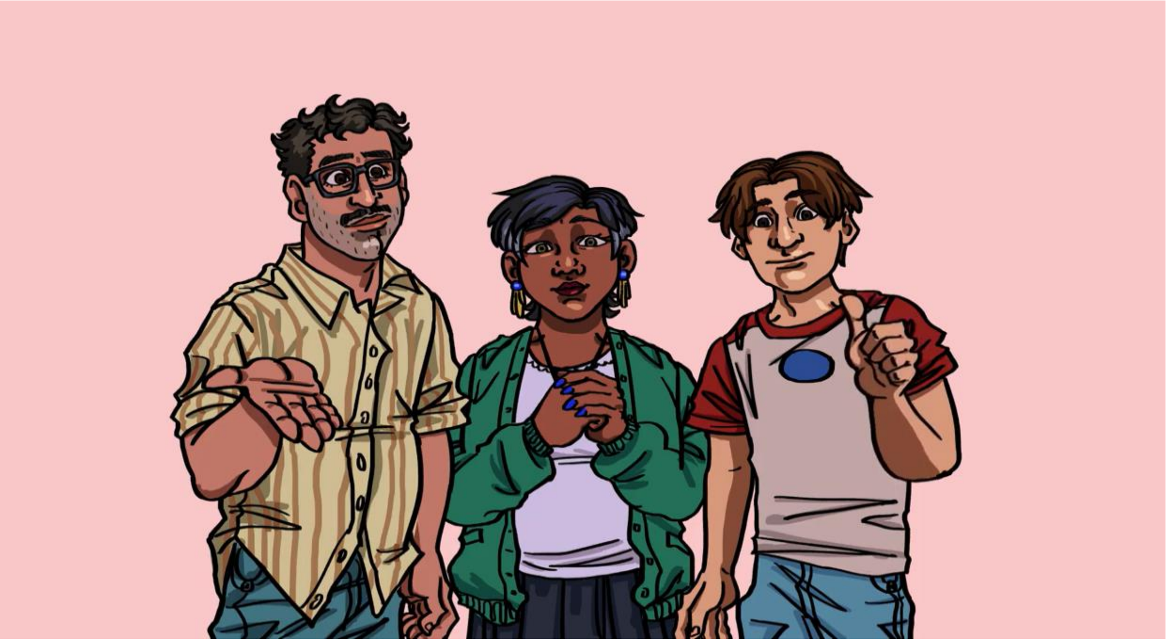

Appendix Figure 10. Screenshot of animated characters from Session 12 (Relapse prevention plan).
