# Supplementary material for: The Efficacy and Usability of an Unguided Web-Based Grief Intervention for Adults Who Lost a Loved One During the COVID-19 Pandemic: Randomized Controlled Trial
Source: J Med Internet Res. 2023 Apr 6;25:e43839. doi: 10.2196/43839 (PMC10131766; doi:10.2196/43839)
Supplement: Multimedia Appendix 3 [file jmir_v25i1e43839_app3.pdf]

This is a Multimedia Appendix to a full manuscript published in the J Med Internet Res. For full copyright and citation information see http://dx.doi.org/10.2196/jmir.43839

Multimedia Appendix 3. Grief COVID web-based intervention. Socio-demographic characteristics of participants

Variable	Treatment	Control	*P* value
	Mdn[IQR] or n(%)	Mdn[IQR] or n(%)	
**Age, years**	46[39-54]	32[25-40]	*<.001*[a]
**Gender**			
Women	63(91.3)	40(88.9)	.57[b]
Men	5(7.2)	5(11.1)	
Nonbinary	1(1.4)	0(0)	
**Country of residence**			
Mexico	44(97.8)	66(95.7)	.48[b]
Colombia	1(2.2)	0(0)	
Other<br>(Honduras, Spain, USA)	0(0)	3(4.2)	
**Working**	48(69.6)	29(64.4)	.57[b]
**Educational attainment**			
High school	7(15.6)	9(13.0)	.97[b]
University- Bachelor's Degree	26(57.8)	44(63.8)	
University- Master's Degree	10(22.2)	14(20.3)	
University- Ph.D.	1(2.2)	1(1.4)	
Other	1(2.2)	1(1.4)	

[a]*P* value for the Mann-Whitney U test

[b]*P* value for the Pearson's χ^2 test
